# E2F6/KDM5C promotes SF3A3 expression and bladder cancer progression through a specific hypomethylated DNA promoter

**DOI:** 10.1186/s12935-022-02475-4

**Published:** 2022-03-05

**Authors:** Kai-Long Liu, Yue-Wei Yin, Bao-Sai Lu, Ya-Lin Niu, Dan-Dan Wang, Bei Shi, Hong Zhang, Ping-Ying Guo, Zhan Yang, Wei Li

**Affiliations:** Department of Urology, The Second Hospital of Hebei Medical University, 215 Heping West Road, Shijiazhuang, 050000 China

**Keywords:** SF3A3, KDM5C, Hypomethylation, Proliferation, Bladder cancer

## Abstract

**Background:**

Abnormal expression of splicing factor 3A subunit 3 (SF3A3), a component of the spliceosome, has been confirmed to be related to the occurrence and development of various cancers. However, the expression and function of SF3A3 in bladder cancer (BC) remains unclear.

**Methods:**

The SF3A3 mRNA and protein level were measured in clinical samples and cell lines by quantitative real-time PCR, Western blot and immunofluorescence staining. Evaluate the clinical correlation between SF3A3 expression and clinicopathological characteristics through statistical analysis in BC patients. The function of SF3A3 in BC cells was determined in vitro using MTT and colony analysis. Co-immunoprecipitation (CoIP) assay was used to detected E2F6 and KDM5C interaction. Luciferase reporter and chromatin immunoprecipitation (ChIP) were used to examine the relationship between E2F6/KDM5C and SF3A3 expression.

**Results:**

In the present study, we demonstrated that expression of SF3A3 was elevated in BC tissue compared to the normal bladder tissue. Importantly, the upregulation of SF3A3 in patients was correlated with poor prognosis. Additionally, overexpression of SF3A3 promoted while depletion of SF3A3 reduced the growth of BC cells in vivo and in vitro. Data from the TCGA database and clinical samples revealed that hypomethylation of the DNA promoter leads to high expression of SF3A3 in BC tissue. We found that upregulation of lysine-specific demethylase 5C (KDM5C) promotes SF3A3 expression via hypomethylation of the DNA promoter. The transcription factor E2F6 interacts with KDM5C, recruits KDM5C to the SF3A3 promoter, and demethylates the GpC island of H3K4me2, leading to high SF3A3 expression and BC progression.

**Conclusions:**

The results demonstrated that depletion of the KDM5C/SF3A3 prevents the growth of BC in vivo and in vitro. The E2F6/KDM5C/SF3A3 pathway may be a potential therapeutic target for BC treatment.

**Supplementary Information:**

The online version contains supplementary material available at 10.1186/s12935-022-02475-4.

## Background

Bladder cancer (BC) is the most common malignant tumor among urinary system cancer. Statistically, there were 81,400 new cases of BC worldwide and more than 17,980 deaths in 2020 [1]. In China, the incidence and mortality of BC are at the forefront of genitourinary system tumors and are showing an upward trend [2]. More than 90% of BC is urothelial cell carcinoma, whereas squamous cell carcinoma accounts for about 5%, and adenocarcinoma is rare [3]. Approximately 75% of the newly diagnosed cases are non-muscular invasive BC (NMIBC). Treatment methods generally include transurethral bladder tumor resection (TURBT), intravesical chemotherapy, and BCG immunotherapy [4, 5]. About 25% of cases belong to myometrial invasive BC (MIBC), which is highly malignant, prone to lymph node metastasis, and has a low 5-year survival rate [6]. Treatment methods generally include radical total cystectomy and urinary diversion [7]. Although there is some progress in the surgical treatment of BC, immunotherapy, and combined radiotherapy and chemotherapy in recent years, the prognosis of invasive and metastatic BC has not been significantly improved [8]. These poor outcomes are due to insufficient understanding of the biological mechanisms of disease recurrence and progression [9]. Therefore, in-depth molecular mechanism research on genes closely related to its malignant progression can provide new ideas and directions for the diagnosis and treatment of BC.

The spliceosome is a multi-subunit complex composed of small non-coding RNAs (U1, U2, U4, U5, and U6) and a variety of related proteins [10]. Overwhelming evidence suggests that splicing is altered in many human tumors [11]. Previous studies have shown that transcriptional upregulation of several spliceosome components is an important means to ensure accurate pre-mRNA splicing and to protect the growth and survival of cancer cells [12]. Splicing factor 3A subunit 3 (SF3A3) is a component of the spliceosome, and it participates in the splicing of precursor mRNA as a component of the precatalytic spliceosome “B” complex [13, 14]). Studies have found that SF3A3 is involved in the occurrence and development of various tumors. The expression of *YTHDF2* and *SF3A3* are positively correlated, and they play a synergistic role in the progression of hepatocellular carcinoma [15]. SF3A3 mediates cellular stress response to regulate tumor suppressor genes expression and cell death [16]. Recently, Maciej et al. showed that SF3A3 selectively regulates MYC-driven splicing and metabolic reprogramming, and the expression level of SF3A3 affects MYC-induced oncogenesis and breast cancer plasticity. However, the expression and role of SF3A3 in BC are still unclear.

Lysine-specific demethylase 5C (KDM5C), also known as JARID1C, is a member of the SMCY homolog family, which plays a key role in transcriptional regulation by H3K4me2/3 demethylation [17]. Studies have shown that abnormal expression of KDM5C is closely related to the occurrence and development of various cancers [18, 19]. Overexpression of KDM5c in human colon cancer cells leads to weakened *FBXW7* transcription and increases c-Jun protein, resulting in the proliferation of colon cancer cells [20]. In addition, KDM5C, which is highly expressed in PCa and CRPC, promotes the proliferation of castration-resistant prostate cancer (CRPC) cells through epigenetic inhibition of phosphatase and tensin homolog (PTEN) genes [21]. It is reported that KDM5C activates estrogen receptor alpha (ERα) target genes while inhibiting type I interferon (IFN) and interferon stimulating genes to promote the growth of ERα-positive breast cancer cells and tumorigenesis [22]. Despite KDM5c is associated with a variety of cancer types, the function of KDM5c in the progression of BC is largely unknown.

In this study, we demonstrated that upregulation of SF3A3 in BC tissue was correlated with poor survival in BC patients. Moreover, overexpression of SF3A3 promoted while depletion of SF3A3 reduced the growth of BC cells. In addition, we found that hypomethylation of the DNA promoter leads to high expression of SF3A3 by upregulating KDM5C. Interestingly, we determined that E2F6 binds to KDM5C and recruits KDM5C to the GpC island of the SF3A3 promoter to induce histone demethylation. Depletion of the KDM5C/SF3A3 prevents BC progression. The E2F6/KDM5C/SF3A3 pathway may be a potential therapeutic target for BC treatment.

## Methods

### Samples collection

The human primary BC tissue and corresponding normal bladder tissue were collected from BC patients in the Second Hospital of Hebei Medical University from July 2017 to June 2021. All BC patients underwent curative radical cystectomy in urology department. The research protocol and sample collection were approved by the Ethics Committee of the Second Hospital of Hebei Medical University, and each patient signed a written informed consent [26].

### Cell culture and transfection

UM-UC-3, 253 J, T24 and J82 cell lines were purchased from The Global Bioresource Center (ATCC, Maryland), and expanded and stored in our laboratory. The above cell were cultured with dulbecco’s modified eagle medium (DMEM) (Gibco, USA) medium supplemented with 10% fetal bovine serum (Clark Bio, Claymont,DE, USA) and 1% penicillin/streptomycin (Gibco, USA). Cells were cultured under humidified conditions of 95% air and 5% CO_2_. Cells were passaged at about 80% confluence. Vectors were transfected into the cells by using Lipofectamine 2000 (Invitrogen) according to the manufacturer’s protocol [23]. The siRNAs such as shSF3A3, shKDM5C and shE2F6 and negative controls were synthesized by GenePharma Co., Ltd. SF3A3 (oeSF3A3), oeKDM5C, and oeE2F6 plasmids and lentiviral vectors were gained from GENEWIZ (Suzhou, China).

### RNA isolation and RT-qPCR

The tissues or cells were lysed by using QIAzol Lysis Reagent (iagen), and then miRNeasy Mini Kit was used to isolation total RNA (217,004; Qiagen). NanoDrop 2000 (Thermo) was used for total RNA quality examination. First strand of cDNA was synthesized by using M-MLV First Strand Kit (Life Technologies) and random hexamer primers. Platinum SYBR Green qPCR Super Mix UDG kit (Invitrogen) were used to detect gene mRNA expression in the ABI 7500 FAST system (Life Technologies). The relative expression levels were normalized to GAPDH and results were analyzed by the 2^−ΔΔCt^ formula [24]. The corresponding primers were listed in Additional file 1: Table S1.

### Western blot

RIPA lysis buffer was used to extraction the proteins from cultured cells and frozen tissue clinical samples. Protein samples was separated in SDS-PAGE and then electrotransferred to PVDFmembrane (Millipore). The membrane was incubated with 5% skimmed milk for 2 h and treatment with the primary antibody overnight at 4 °C. The antibodies in this study as follows: anti-β-actin (1:1000, sc-47,778), anti-SF3A3 (1:1000, ab176581), anti-c-MYC (1:500, ab32072), anti-KDM5C (1:1000, ab259913), and anti-E2F6 (1:500, ab155978). Next day, HRP-conjugated secondary antibody (1:5000, Rockland) was used to reaction with the membrane. Western blots were processed by ECL (enhanced chemiluminescence, Millipore) and detected using Fuazon Fx (Vilber Lourmat). FusionCapt Advance Fx5 software (Vilber Lourmat) were used to capture and process images [25] (Additional file 2).

### Vector construction and luciferase reporter assay

The 2-kb SF3A3 promoter sequence was inserted into the pGL3 basic vector after treatment with Mlu1 and Xho1. Sanger sequencing was used to confirm the sequence. BC cell lines were seeded into a 24-well plate, and SF3A3-prom vector, oeKDM5C, or shE2F6 and the corresponding control vector were co-transfected to cells for 24 h. Luciferase activity was measured by Dual-Glo Luciferase Assay System (Promega, Madison, WI, USA) with Flash and Glow (LB955, Berthold Technologies) [26].

### Xenograft animal model

Approximately 18–22 g male BALB/c nude mice (4–6 weeks old) were purchased from Vital River Laboratory Animal Technology Co., Ltd. (Beijing) and used for Xenograft animal mode. A total of 5 × 10^6^ T24 cells stable shSF3A3/shKDM5C-infected were collected and resuspended in PBS and mixed with 50% Matrigel (BD) to form a volume of 0.2 mL Inject the mixed suspension subcutaneously into the right dorsal side. One week later, the length and width of the mouse tumors were measured twice a week. Then, we used the following formula to calculate the tumor volume: tumor volume = (length × width 2)/2. After 28 days, the mice were euthanized by carbon dioxide asphyxiation [23, 25, 27]. The euthanasia protocol is as described previously [28]. The mice were placed in a transparent airtight box (20 cm × 12 cm × 12 cm). A concentration of 99.9% carbon dioxide was injected into the closed box at a flow rate of 0.6 L/min (approximately 20% of the box volume). The mice were exposed to the carbon dioxide until a complete cessation of breathing, and observed for at least 2 min (usually about 5–10 min in total). Then, the mice were taken out of the box and treated with cervical dislocation to ensure death. Finally, xenograft tumor tissue were removed for later use.

### Morphometry and histology

Morphometry and histology were detected by hematoxylin and eosin staining as described before [27, 29]. BC and corresponding normal tissues were fixed in formalin solution and then embedded in conventional paraffin. 5 μm thick sections were used for hematoxylin and eosin staining or immunofluorescence staining. A Leica microscope (Leica DM6000B, Switzerland) was used to acquire sectional images, and LAS V.4.4 (Leica) was used for digitization.

### Immunofluorescence staining

The above-mentioned embedded clinical sample tissue and xenograft tissue were sliced and routinely deparaffinized to water as described before [30]. The tissue sections were blocked with goat serum after antigen retrieval. These were incubated overnight with anti-SF3A3 (ab176581) and anti-c-MYC (1:500, ab32072). The tissue was treated with fluorescein-labeled anti-rabbit IgG (KPL) and rhodamine-labeled anti-mouse IgG (KPL) [30]. DAPI (157,574; MB Biomedical) was used for nuclear staining. The images were captured using a confocal microscope (DM6000 CFS; Leica) and processed using LAS AF software [27, 29].

### MTS assay

According to the product’s instruction manual (ab197010), the cell viability was examined by the MTS. In brief, T24 and J82 cells were planted into 96-well plates and transfected with the corresponding vectors. Then, 20 µL of MTS reagent were added to each well and incubated for 0.5–4 h. Finally, the absorbance of the cells was detected by a microplate reader at 490 nm (Thermo Fisher, USA).

### Chromatin immunoprecipitation (ChIP) assay

In order to explore the interaction of transcription factors with DNA, we performed chromatin immunoprecipitation (ChIP) as described above [31]. Cultured T24 cells were fixed with formaldehyde and neutralized with glycine to prepare cross-linked chromatin. After that DNA samples were sonicated to an average long of 400–600 bp. These samples were diluted and then incubated with protein A-Sepharose/Salmon sperm DNA for at 4 °C. Then anti-KDM5C, anti-E2F6, or anti-IgG (as a negative control) antibody were used to immunoprecipitate DNA fragments. After the crosslinking was reversed, the binding rate of KDM5C and E2F6 on the SF3A3 promoter was checked. The result was confirmed by qRT-PCR.

### Colony formation assay

In order to detected the cell proliferation the colony formation assay was performed [30]. 100 cells/well were seeded into a six-well plate and continuous cultured for 10–14 days. Then, these were fixed in methanol solution and colony was stained with 0.5% crystal violet. The number of colonies was counted under the microscope.

### Co-immunoprecipitation (CoIP) assay

Cell lysates were immunoprecipitated by overnight incubation with the indicated antibody at 4 °C, followed by another overnight incubation with protein A-agarose for 1 h. The protein A-agarose-antigen-antibody complexes were collected through centrifugation at 12,000*g* for 2 min at 4 °C, followed by washing five times with 1 mL of immunoprecipitation-HAT buffer for 20 min each time at 4 °C [24]. The bound proteins were resolved on an SDS-PAGE gel, followed by Western blotting using the corresponding antibody.

### Statistical analysis

Statistical analysis as mentioned before [24]. Data were expressed as mean ± SEM. The Student’s t-test was used to analyze the difference between the two groups. Spearman correlation analysis was used to evaluate correlation analysis. A value of P < 0.05 was considered statistically significant. GraphPad Prism 7.0 software was used for statistical analysis (GraphPad Software) [30].

## Results

### The upregulation of SF3A3 contributes to poor prognosis of BC patients

**Fig. 1:**
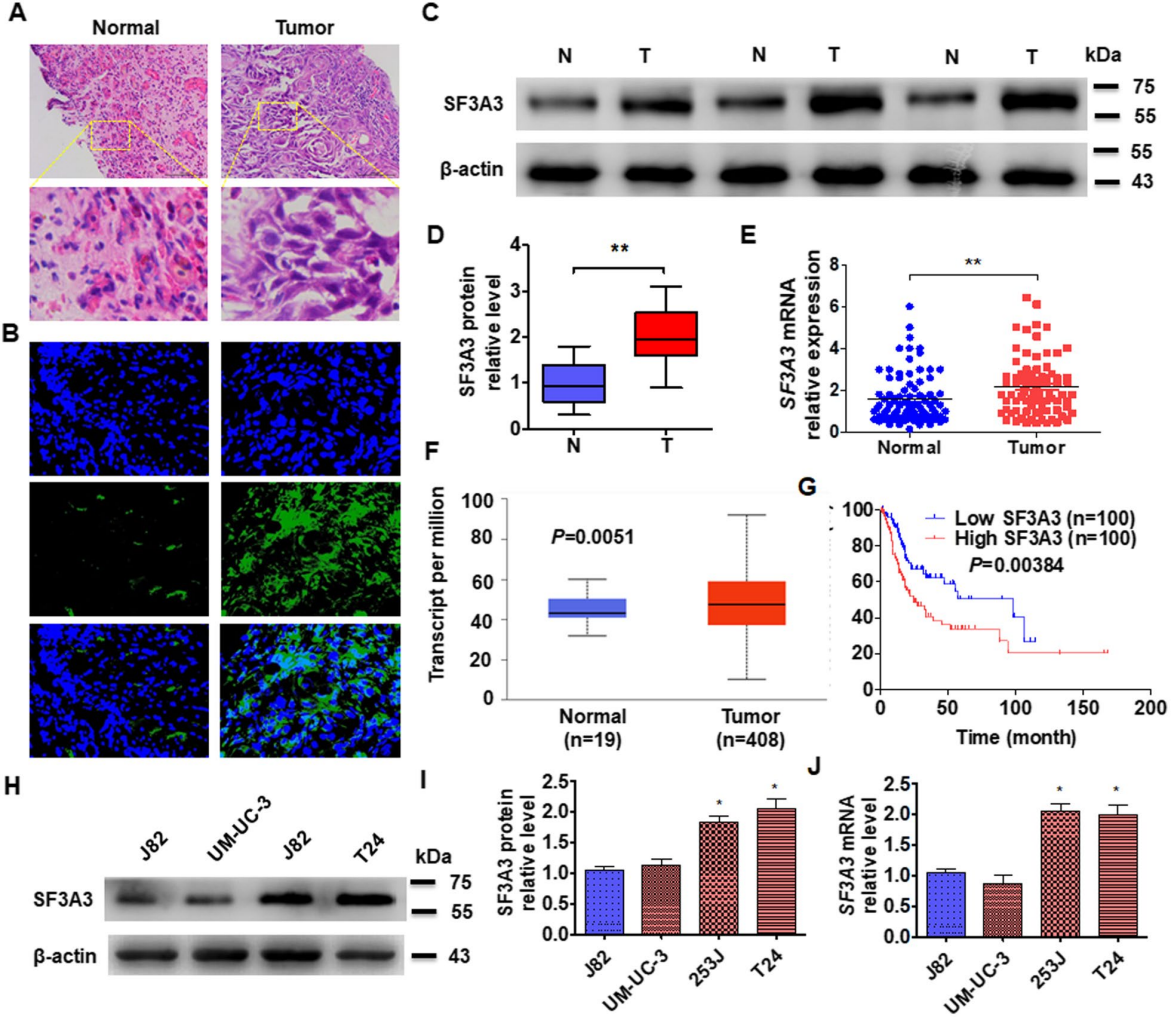
SF3A3 is upregulated and correlates with poor survival with bladder cancer patients. **A** Hematoxylin and eosin staining in tumor and normal bladder tissues. Scale Bar = 25 μm. **B** Immunofluorescence staining detects the expression of SF3A3 in normal and bladder cancer tissues. Bar = 50 μm. **C** The SF3A3 protein levels were detected by Western blot in tumor (T) and normal (N) bladder tissues. **D** Quantitative analysis of protein expression in **C**. **E** RT-qPCR was used to detect the mRNA level of SF3A3 in normal (n = 49) and tumor (n = 49) bladder tissues. **F** The data of SF3A3 expression was analyzed from the TCGA database (http://ualcan.path.uab.edu/analysis.html) in tumor and normal bladder tissues. **G** The overall survival of BC patients was analyzed from the data of TCGA with low (n = 100) and high (n = 100) SF3A3 levels by Kaplan–Meier analysis (http://www.oncolnc.org/). **H** SF3A3 protein level was measured by Western blot in BC cell lines (J82, UM-UC-3, 253 J, and T24). **I** Quantitative analysis of proteins level of **H**. **J** RT-qPCR was used to examine the mRNA level of SF3A3 in different cell lines. All data are expressed as mean ± SEM and come from three independent experiments. *P < 0.05, **P < 0.01 vs. the corresponding controls

A previous study showed that SF3A3 facilitates breast cancer progression [32]. To explore the expression of SF3A3 in BC tissues, we first collected the clinical samples and confirmed tumor and normal tissues by using hematoxylin and eosin staining (Fig. 1A). Then, we examined the expression of SF3A3 in BC tissues by immunofluorescence staining. The results showed that the fluorescence intensity of SF3A3 significantly increase in BC tissue (Fig. 1B). The Western blot results also revealed that the SF3A3 protein level was significantly increased in BC tissues (Fig. 1C, D). By analyzing the mRNA expression in the clinical sample or TCGA data set, we demonstrated that expression of SF3A3 was markedly upregulated in BC tissue than in normal bladder tissue (Fig. 1E, F). In addition, the Kaplan–Meier correlation analysis in TCGA database also showed that patients with high SF3A3 expression in BC were predicted to have poorer overall survival (Fig. 1G). Furthermore, the clinicopathological factors of SF3A3 expression level were positively correlated with tumor size but did not correlate with other such as age, gender, and tumor grade (Table 1). Next, we then measured the expression of SF3A3 in BC cell lines and found that SF3A3 had a higher expression in T24 cells and lower expression in J82 cells (Fig. 1H–J). These results confirmed that SF3A3 is elevated in BC tissue and may be associated with BC progression.

**Table 1:** The SF3A3 expression and clinicopathological characteristics

Characteristic	Number of patients (%)	SF3A3 expression		P value^a^
		Low (%)	High (%)	
Patients	82	42	40	
Age (years)				
≤ 60^b^	34	18 (52.86)	16 (47.14)	0.826
> 60	48	24 (50.55)	24 (49.45)	
Gender				
Male	62	30 (54.90)	32 (45.10)	0.445
Female	20	12 (45.76)	8 (54.24)	
Tumor size (cm)				
≤ 3.0^c^	54	31 (54.72)	13 (45.28)	**0.014**
> 3.0	28	11 (27.27)	17 (72.73)	
Tumor grade				
Low	49	23 (46.39)	26 (53.61)	0.376
High	33	19 (56.25)	14 (43.75)	
T classification				
Ta, T1	53	31(43.81)	22 (56.19)	0.106
T2–T4	29	11(60.71)	18 (39.29)	
pN status				
pN−	58	32 (48.25)	26 (51.75)	0.334
pN+	24	10 (61.70)	14 (38.30)	
Tumor multiplicity				
Unifocal	35	14 (40.58)	21 (59.42)	0.118
Multifocal	47	28 (42.39)	19 (57.61)	

Significant associations are shown in bold in the p value column (p value < 0.05)

^a^Chi-square test

^b^Median age

^c^Median size

### SF3A3 facilitates the growth of BC cells in vitro

**Fig. 2:**
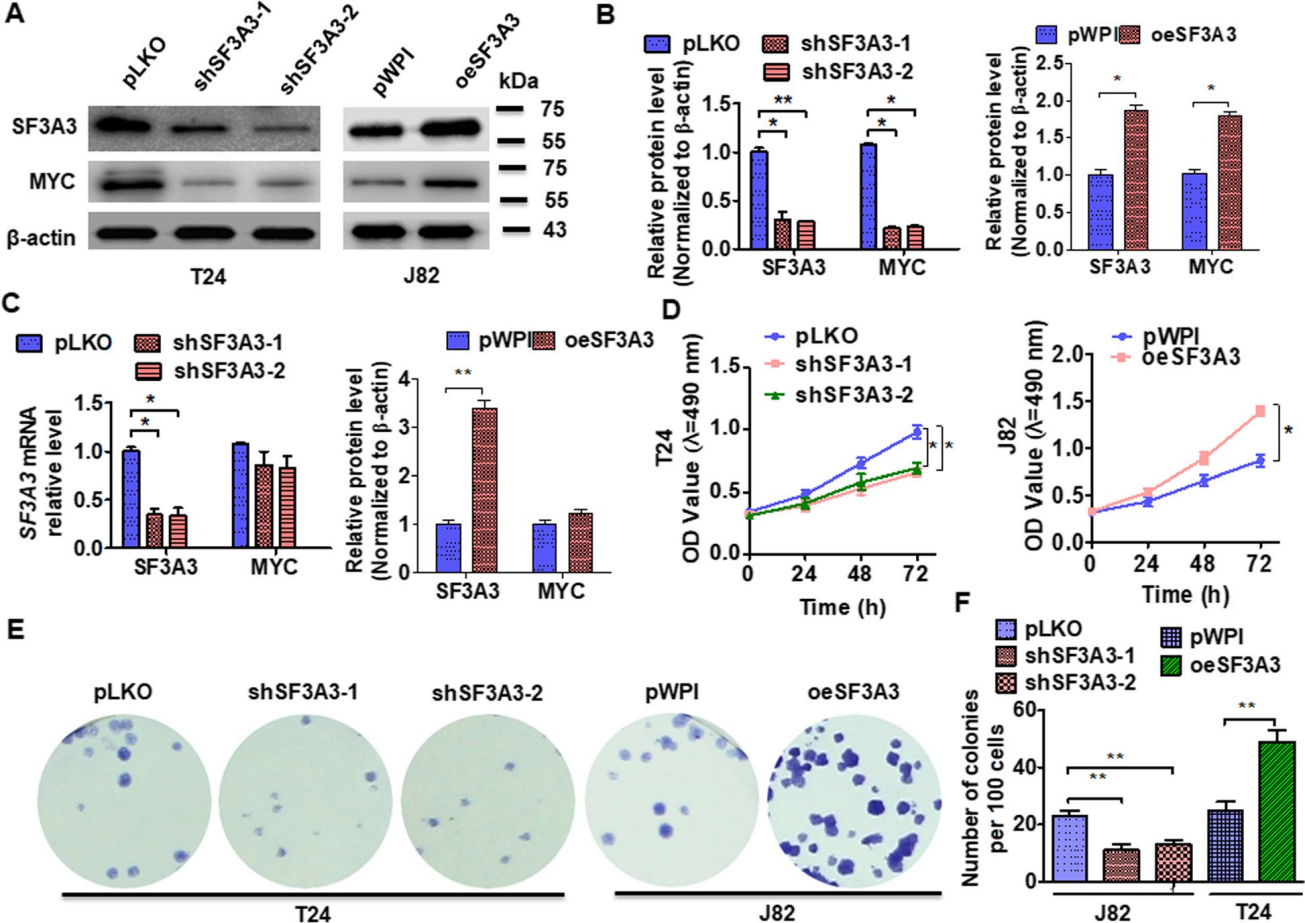
SF3A3 facilitates the proliferation of bladder cancer cells in vitro. **A** T24 cells were transfected with pKLO, shSF3A3-1, shSF3A3-2, or J82 with pWPI and oeSF3A3 vectors, and SF3A3 and MYC proteins levels were measured by using Western blot. **B** Quantitative analysis of protein expression in **A**. **C** T24 and J82 cells transfected as A and SF3A3, and MYC mRNA expression was examined by RT-qPCR. **D**–**F** T24 and J82 cells were transfected as in **A**, and then cell viability was explored by MTS (**D**) and colony formation assays (**E**, **F**). All data are expressed as mean ± SEM and come from three independent experiments. *P < 0.05, **P < 0.01 vs. the corresponding controls

To explore the function of SF3A3 in BC, we first constructed two short hairpin RNA (shRNA) targeting SF3A3 and knocked it out in T24 cells while overexpressing SF3A3 in J82 cells by transfecting with the pWPI-SF3A3 vector. The results showed that shSF3A3 RNAs transfection significantly downregulated while pWPI-SF3A3 vector transfection upregulated the SF3A3 and MYC protein expression but did not affect MYC mRNA level (Fig. 2A–C). Next, we performed the MTS and colony formation assay to measure cell viability. The results showed that depletion of SF3A3 significantly reduced T24 cell proliferation while overexpression of SF3A3 promoted cell growth (Fig. 2D–F). Together, these data reveal a function of SF3A3 in the regulation of cell growth in BC.

### Upregulated KDM5C promotes SF3A3 expression via hypomethylation of the DNA promoter

**Fig. 3:**
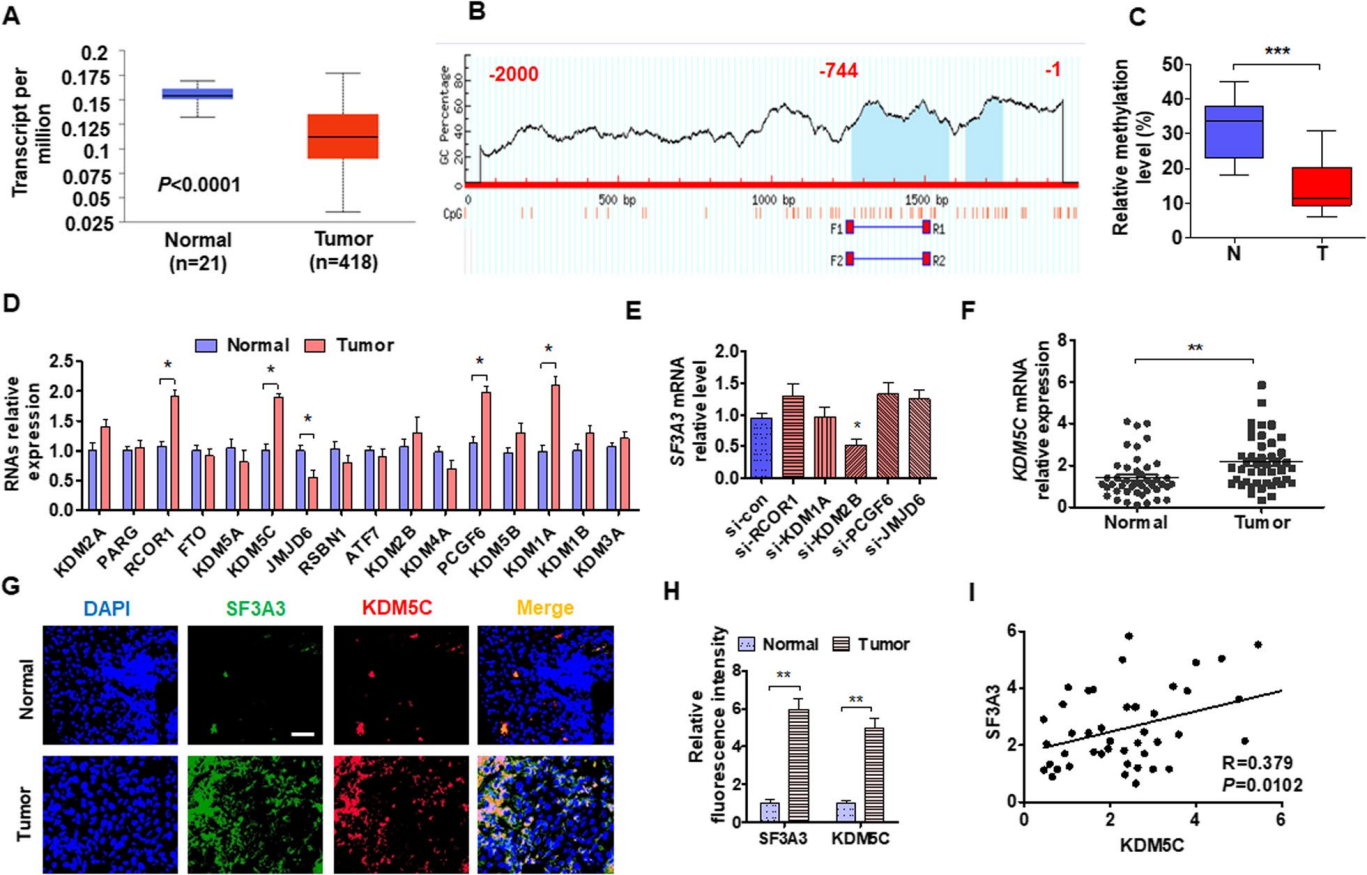
Hypomethylation of promoter induces SF3A3 expression by upregulating KDM5C. **A** The DNA methylation level of SF3A3 promoter was explored from the TCGA database (http://ualcan.path.uab.edu/analysis.html) in tumor and normal bladder tissues. **B** The GpC island of SF3A3 promoter was analyzed by Methprimer (http://www.urogene.org/cgi-bin/methprimer/methprimer.cgi). **C** DNA methylation was measured using bisulfite sequencing PCR (BSP) in the GpC island of the SF3A3 promoter. **D** The expression of candidate DNA demethylases was measured by RT-qPCR in tumor and normal bladder tissues. **E** T24 cells were transfected with indicated siRNA, and then SF3A3 mRNA was detected by RT-qPCR. **F** RT-qPCR was used to explore the KDM5C expression in tumor and normal bladder tissues. **G** Double immunofluorescence staining was used to examine the expression of SF3A3 and KDM5C in tumor and normal bladder tissues. **H** Quantitative analysis of the fluorescence intensity SF3A3 and KDM5C. **I** Analysis of the correlation between SF3A3 and KDM5C expression in BC. **J** The overall survival of BC patients was analyzed with KDM5C levels by Kaplan–Meier analysis from the data of TCGA (http://www.oncolnc.org/). All data are expressed as mean ± SEM and come from three independent experiments. *P < 0.05, **P < 0.01 vs. the corresponding controls

Studies showed that methylation dysfunction of gene promoters induces tumor progression [33, 34]. To investigate whether abnormal expression of SF3A3 is regulated by DNA methylation, we first analyzed methylation modification of SF3A3 promoter from TCGA database and found that there is hypomethylation in SF3A3 promoter compared with normal bladder tissue (Fig. 3A, Additional file 2). Then, we analyzed the GpC island of SF3A3 promoter (Fig. 3B) and examined the methylation level in GpC island using MSP and bisulfite sequencing PCR (BSP) analyses. The results revealed that the methylation level of SF3A3 CpG was lower in BC than that in normal bladder tissue (Fig. 3C). To investigate which demethylases may involve in SF3A3 promoter hypomethylation, we explored candidate genes in the clinical samples. As shown in Fig. 3D, *KDM5C*, *KDM2B*, and *PCGF6* were upregulated and *JMJD6* was downregulated in BC tissue. Next, siRNAs of these genes were synthesized and transfected into T24 cells, and then the expression of SF3A3 was examined by RT-qPCR. As showed in Fig. 3F, only knocking out *KDM5C* reduced SF3A3 expression in T24 cells. In addition, we detected the expression of SF3A3 in clinical samples and found that the expression of SF3A3 was increased in tumor tissue (Fig. 3F). Similar results were found in immunofluorescence staining (Fig. 3G, H). Otherwise, SF3A3 is also highly expressed in cells with high *KDM5C* expression (Fig. 3G). Furthermore, correlation analysis revealed that SF3A3 mRNA level was positively correlated with *KDM5C* expression in BC tissue (Fig. 3I). Besides, the high expression of *KDM5C* in BC with patients was associated with a poor prognosis (Fig. 3J). These findings suggest that SF3A3 is a regulator modulated by *KDM5C* to drive tissue expansion.

### E2F6 interaction with KDM5C and boosts histone demethylation

**Fig. 4:**
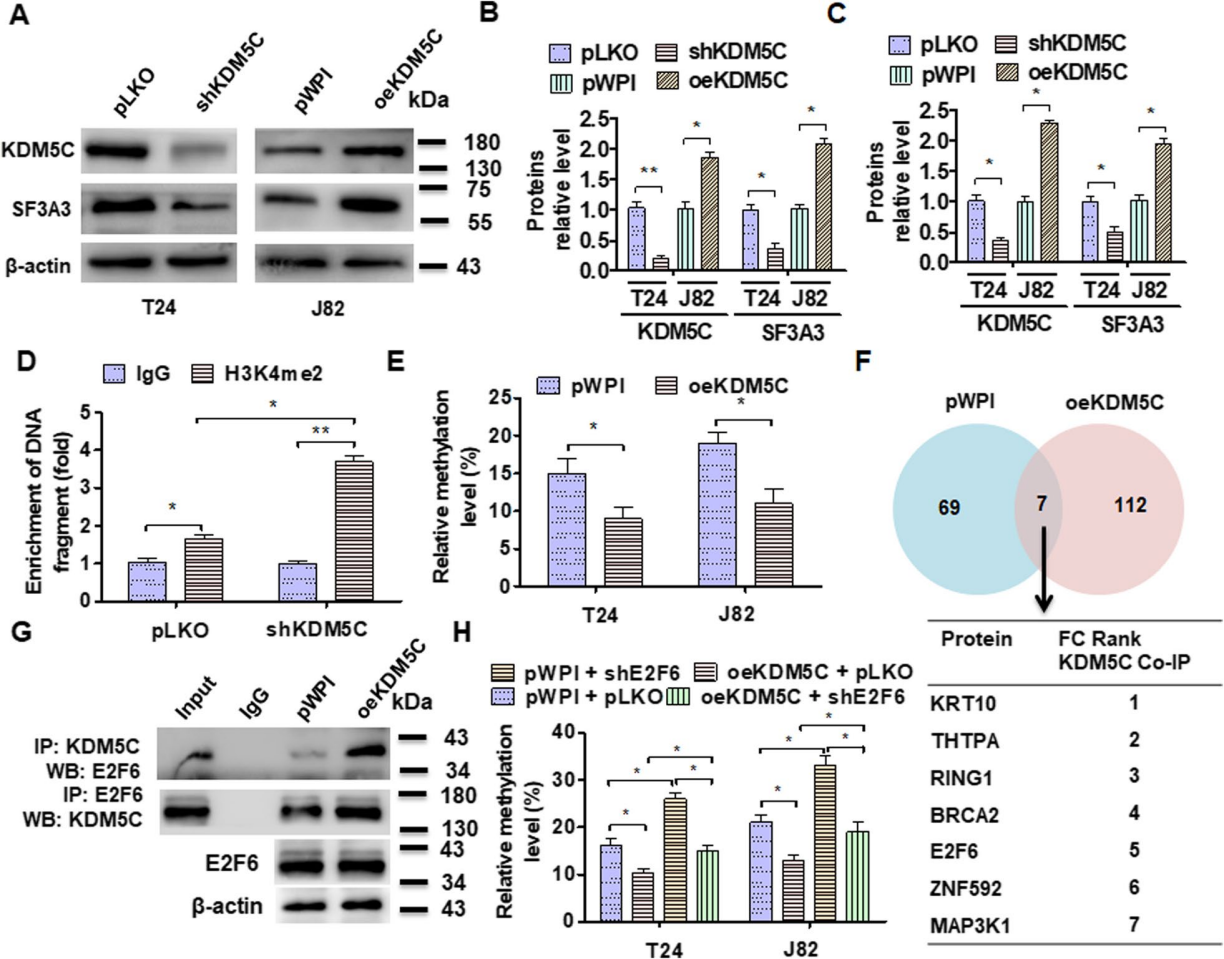
E2F6 recruits KDM5C to the GpC island of the SF3A3 promoter and induces histone demethylation. **A** T24 cells were transfected with pKLO, shKDM5C, or J82 with pWPI and oeKDM5C vectors, and KDM5C and SF3A3 proteins level were measured using Western blot. **B** Quantitative analysis of protein expression in **A**. **C** T24 and J82 cells were transfected as in A and KDM5C, and SF3A3 mRNA expression was examined by RT-qPCR. **D** ChIP-PCR with H3K4me2 or IgG detected the CpG isolate of SF3A3 promoter in pWPI or oeSF3A3 vectors transfected-J82 cells. **E** T24 and J82 were transfected with pWPI or oeKDM5C vectors, and then, bisulfite sequencing PCR (BSP) was used to examine the DNA methylation in the GpC island of the SF3A3 promoter. **F** J82 cells were transfected with pWPI or oeKDM5C and a coimmunoprecipitation coupled with mass spectrometry (CoIP-MS) was performed with KDM5C antibody. The down table exhibits 7 proteins that increased interaction with KDM5C after overexpression of KDM5C. **G** CoIP-Western blot detected the interaction between KDM5C and E2F6 after KDM5C overexpression. **H** T24 and J82 were transfected as in **A** and then DNA methylation condition was measured using BSP in the GpC island of SF3A3 promoter. All data are expressed as mean ± SEM and come from three independent experiments. *P < 0.05, **P < 0.01 vs. the corresponding controls

T figure out whether *KDM5C* regulates SF3A3 expression by demethylating, we first overexpressed or depleted *KDM5C* in cells and examined this gene expression. As shown in Fig. 4A, B, depletion of *KDM5C* reduced the expression of KDM5C and SF3A3 in T24 cells while overexpression of *KDM5C* upregulates both proteins level. Similar results were found in RT-qPCR (Fig. 4C). To investigate whether KDM5C regulates the histone methylation level, we transfected with oeKDM5C and detected the H3K4me2 methylation in the GpC island of SF3A3 promoter by ChIP. As expected, depletion of KDM5C significantly elevated the H3K4me2 methylation level in T24 cells (Fig. 4D). Conversely, overexpression of KDM5C markedly depressed the methylation level in the GpC island of the SF3A3 promoter (Fig. 4E). To identify how KDM5C regulated the methylation of SF3A3 specifically, we performed a CoIP -mass spectrometry (CoIP-MS). Seven proteins were found to enhance the interaction with KDM5C in KDM5C-overexpressing BC cells (Fig. 4F). CoIP coupled with Western blot confirmed that E2F6 interacted with KDM5C (Fig. 4G). Additionally, knocking out E2F6 would disrupt the methylation of the SF3A3 promoter, which was induced by overexpressing KDM5C in BC cells (Fig. 4H). Collectively, these data revealed that a function of KDM5C is interaction with E2F6 and promotion of SF3A3 expression via histone demethylation.

### E2F6/KDM5C binds to the GpC island of the SF3A3 promoter and facilitates the growth of BC cells in vitro

**Fig. 5:**
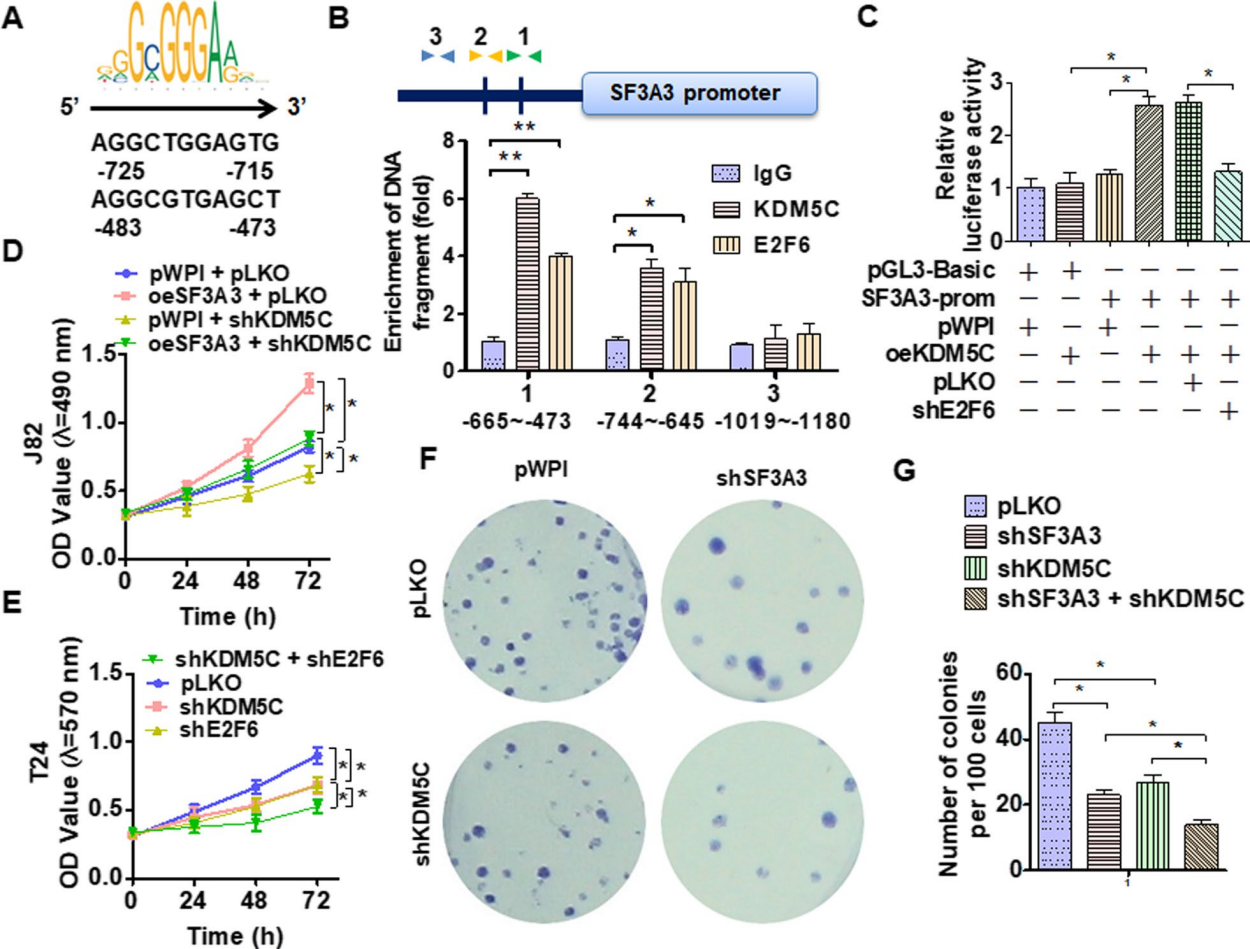
E2F6/KDM5C-regulated SF3A3 expression facilitates the growth of BC cells. **A** We predicted the potential E2F6 binding site within the GpC island of SF3A3 promoter using the Ensembl and PROMO 3.0 websites. **B** ChIP-PCR was used to explore the binding site of E2F6 in GpC island with KDM5C and E2F6 antibodies. **C** Luciferase reporter assays were performed in T24 cells that were co-transfected with indicated vectors. **D**, **E** J82 and T24 cells were transfected with indicated vectors, and then, cell viability was measured by MTS assay. **F** Cell proliferation was measured by colony formation assays in J82 cells after transfection with shSF3A3 or shROCR1 or both together. **G** Quantitative analysis of **F**. All data are expressed as mean ± SEM and come from three independent experiments. *P < 0.05, **P < 0.01 vs. the corresponding controls

To investigate whether transcription factor E2F6 recruits KDM5C to GpC island of the SF3A3 and regulates SF3A3 expression, we first analyzed the potential E2F6 binding site within the GpC island of SF3A3 promoter. As shown in Fig. 5A, there are two potential E2F6 binding elements. Next, ChIP analysis was performed and demonstrated that E2F6/KDM5C bound to the GpC island located – 744 to – 665 bp within the SF3A3 promoter (Fig. 5B). To further confirm whether E2F6/KDM5C regulates the promoter activity of SF3A3, we performed the luciferase assay in J82 cells. The results revealed that KDM5C overexpression enhanced the luciferase activity of the SF3A3 promoter and this would be reversed by depleting GF1B simultaneously (Fig. 5C). These results revealed that E2F6/KDM5C binds to the GpC island of the SF3A3 promoter and regulates SF3A3expression. To examine the function of E2F6/KDM5C in BC cells, we performed rescue experiments. Overexpression of SF3A3 promoted the J82 cell growth and this would be reversed with depletion of KDM5C (Fig. 5D). In addition, knocking out *KDM5C* or *E2F6* alone could depress the proliferation of T24 cells and depletion of both will enhance this effect (Fig. 5E). In parallel, the colony formation assays provided similar results (Fig. 5F, G). Collectively, these data suggest that E2F6/KDM5C regulates cell growth by binding to the SF3A3 promoter.

### Interference with the E2F6/KDM5C/SF3A3 axis prevents the growth of BC xenografts in vivo

**Fig. 6:**
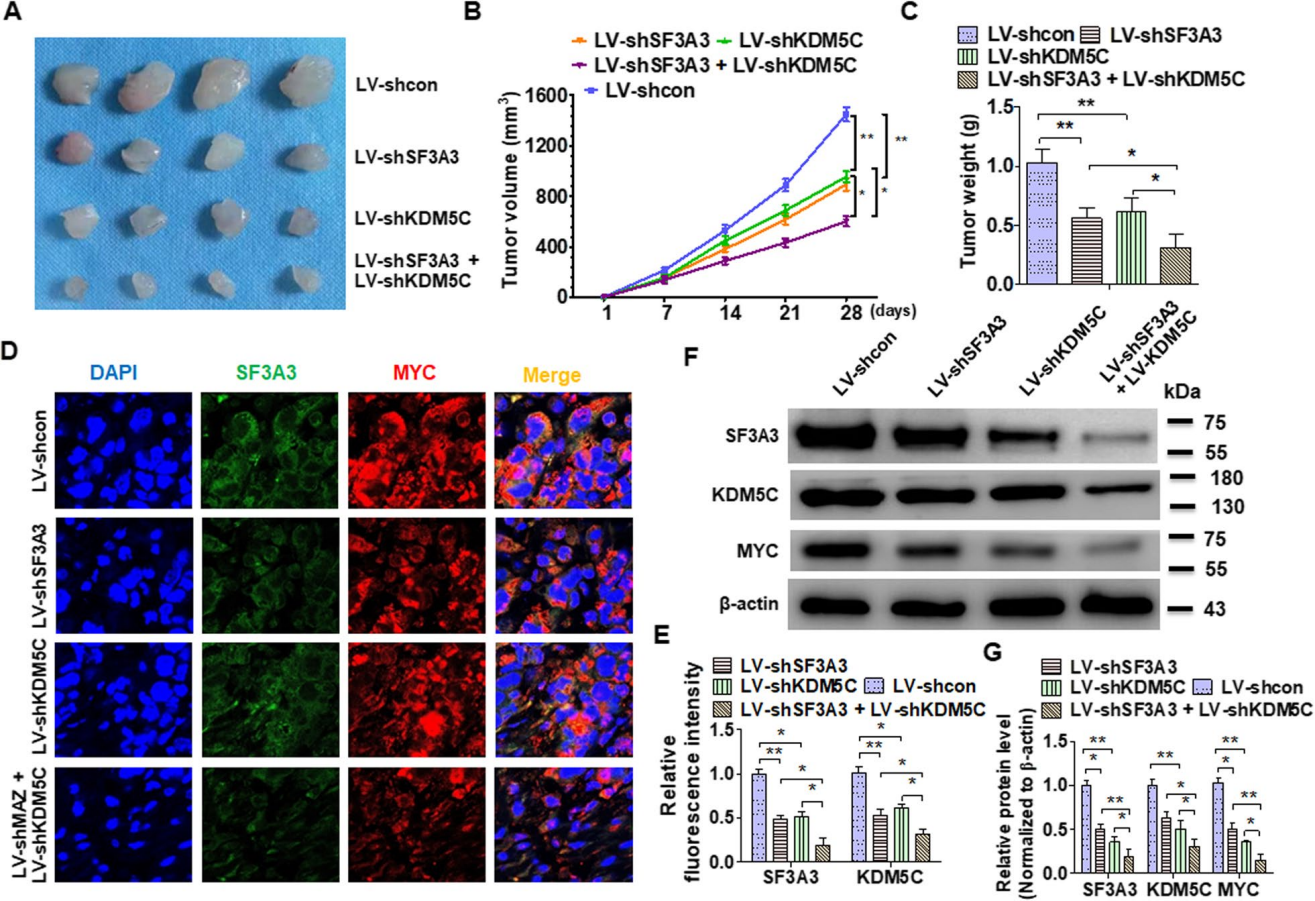
The disruption of the E2F6/KDM5C/SF3A3 axis inhibits BC xenograft progression in vivo. **A** T24 cells were stably depleted of SF3A3, KDM5C, or both and then injected into nude mice to construct BC xenograft tumors. Tumor volumes were displayed by direct measurement. **B** Tumor sizes were shown in each group of mice. **C** The wet weight of xenograft tumors in each group of mice was analyzed. **D** Double immunofluorescence staining was used to detect SF3A3 and MYC expression in xenograft tumor tissues. **E** Quantitative analysis of the fluorescence intensity SF3A3 and KDM5C. **F** The SF3A3, KDM5C, and MYC proteins levels were examined in xenograft tumors using Western blotting. **G** Quantitative analysis of **F**. All data are expressed as mean ± SEM and come from three independent experiments. *P < 0.05, **P < 0.01 vs. the corresponding controls

To study whether disruption of the E2F6/KDM5C/SF3A3 axis inhibits cell proliferation in vivo, a xenografts model was performed. As shown in Fig. 6A, knocking out SF3A3 or KDM5C alone results in smaller tumors compared to injection of control vector cells. In addition, the tumor volume and wet weights were much lower in mice implanted with SF3A3/KDM5C double depletion cells than that only single knocked-out cells (Fig. 6B, C). Double immunofluorescence staining from the tumor tissue demonstrated that depletion of either SF3A3 or KDM5C showed significantly reduced fluorescence intensity compared to control cells while this decrease was enhanced by deletion of both SF3A3 and KDM5C, simultaneously (Fig. 6D, E). Consistent with these, Western blot analysis of tumor tissue also yielded similar results (Fig. 6F, G). Taken together, our combined results indicated that blocking the E2F6/KDM5C/SF3A3 axis inhibits the growth of BC xenografts in vivo.

## Discussion

In the present study, we identified an E2F6/KDM5C/SF3A3 axis that drives BC progression. Firstly, SF3A3 expression was significantly elevated in BC tissue compared to normal bladder tissue, and upregulation of SF3A3 in patients was correlated with poor prognosis. Secondly, overexpression of SF3A3 promoted and depletion of it inhibited BC cell proliferation in vivo and in vitro. Thirdly, the high expression of SF3A3 is caused by the hypomethylation of the DNA promoter driven by the upregulation of KDM5C in BC tissue. In addition, transcription factor E2F5 bound and recruited KDM5C to the GpC island of the SF3A3 promoter and then promoted demethylation of H3K4me2. These findings suggest that the RE2F6/KDM5C/SF3A3 axis is a critical promoter of BC initiation and progression.

DNA methylation is one of the important chemical modifications of DNA involved in gene expression programming [35]. A large number of studies have shown that one of the hallmarks of cancer is abnormal DNA methylation [36]. In the past 20 years, the main research focus in this field has been the hypermethylation of tumor suppressor genes, but there is a relative lack of screening and research on hypomethylation genes for different cancers [37]. KDM5 family proteins include 4 subtypes of KDM5A/B/C/D, which catalyze the detrimethylation and dimethyl labeling of lysine 4 on histone H3 (H3K4), thereby regulating genes expression [38]. Depending on the methylation site, KDM5 can either activate or inhibit transcription [39]. New evidence suggests that the dysregulation of KDM5 is related to important phenotypic consequences of various types of cancer [40]. For example, KDM5C promotes the proliferation of colon cancer cells through the FBXW7-c-Jun regulatory axis [20]. KDM5c inhibits the multidrug resistance of colon cancer cell lines by down-regulating *ABCC1* [41]. *KDM5C* is transcriptionally regulated by BRD4 and promotes the proliferation of CRPC cells by inhibiting PTEN [21]. However, the expression and function of KDM5C in BC are still unclear. In this study, we found that KDM5C was highly expressed in BC tissue compared to normal bladder tissue, and upregulation of KDM5C promoted the hypomethylation of the DNA promoter in the GpC island of SF3A3. The hypomethylation of this promoter induced expression of SF3A3 and BC progression. Blocking of KDM5C can reduce the cell proliferation of BC in vivo or in vitro.

Human splicing factor SF3A is an important part of 17 S U2 snRNP (small nuclear ribonucleoprotein particles). SF3A interacts with pre-mRNA branch sites to form an early spliceosome [42]. Using RNA to treat HeLa cells, the researchers found that removing individual subunits resulted in overall suppression of splicing, indicating that SF3A is a constitutive splicing factor [43]. SF3A includes three subtypes: SF3A1, SF3A2, and SF3A3. The structure and function analysis has clarified the relationship between the interaction of SF3A heterotrimer and the assembly of U2 snRNP and spliceosome [44]. Previous studies have shown that the transcriptional upregulation of several spliceosome components is an important means to ensure accurate pre-mRNA splicing and protect the growth and survival of cancer cells [12]. Recent studies have shown that the abnormal expression of SF3A3 is closely related to tumor progression [15]. SF3A3 selectively regulates MYC-driven splicing and metabolic reprogramming, which, in turn, induce tumorigenesis and breast cancer plasticity [32]. However, the expression of SF3A3 in BC and its function is largely unknown. In this study, we found that compared with normal bladder tissue, the expression of SF3A3 in BC tissue was significantly increased, and the upregulation of SF3A3 in patients was associated with a poor prognosis. In addition, the overexpression of SF3A3 promotes and deletion of it inhibits BC cell proliferation in vivo and in vitro. Furthermore, we found that the high expression of SF3A3 is caused by the hypomethylation of the DNA promoter driven by the upregulation of KDM5C in BC tissue. Inhibition of either SF3A3 or KDM5C reduced the progression of BC.

In conclusion, the present study reveals that elevation of SF3A3 promotes BC cell growth and is associated with poor BC prognosis. The upregulation of KDM5C was recruited by E2F6 to GpC island of SF3A3 promoter and demethylated H3K4me2, then driving expression of SF3A3 and BC progression. These findings highlight that the E2F6/KDM5C/SF3A3 axis is a potential therapeutic target for BC treatment.

## Supplementary Information

**Additional file 1: Table S1.** Oligos used in the study.

**Additional file 2: Fig. S1.** Analyzed some genes positively related to SF3A3 expression in BC tissues from the TCGA database.

## Data Availability

Not applicable.
