# Supplementary material for: E2F6/KDM5C promotes SF3A3 expression and bladder cancer progression through a specific hypomethylated DNA promoter
Source: Cancer Cell Int. 2022 Mar 5;22:109. doi: 10.1186/s12935-022-02475-4 (PMC8897952; doi:10.1186/s12935-022-02475-4)
Supplement: Supplementary file 1 — Additional file 1: Table S1. Oligos used in the study. [file 12935_2022_2475_MOESM1_ESM.docx]

**Supplementary table 1**  Oligos used in the study

| Name | Sequcence 5’ to 3’ |
| --- | --- |
| SF3A3-F1 | CCATGCAAGATATCTGTGTGCC |
| SF3A3-R1 | TGCTTGTCTCTTTCGGCCAT |
| MYC-F | GCAATGCGTTGCTGGGTTAT |
| MYC-R | CGCATCCTTGTCCTGTGAGT |
| KDM2A-F | GTGACGCAGCAGCATTGTTC |
| KDM2A-R | AACCGTGGAGCTGGTTTCAT |
| PARG-F | AAGCGGCGCATTGAGTTTTC |
| PARG-R | CTGATTCCGCTGTCTTGATTCC |
| RCOR1-F | ATGCCCGGAAACAAAAACGG |
| RCOR1-R | GTTGTCTCAGCACCGTGGTA |
| FTO-F | GGTTGAGTTCGAGTGGCTGA |
| FTO-R | GAAGCAGTTTGCAAGCACGA |
| KDM5A-F | ATGACAATGGTGGACCGCAT |
| KDM5A-R | CGGGCAACTTCTGAAGGGAT |
| KDM5C-F | ATGCCTAAGGTCCAGGGCTT |
| KDM5C-R | GACTCAGGGATGCTGTGGTTC |
| JMJD6-F | TCCACAGGGATAGCTTCCGA |
| JMJD6-R | AGCTTACGCTGAAAGCACCT |
| RSBN1-F | GACATACTGCTGTGGCACCT |
| RSBN1-R | TGGTCCGAGGTAGGTACTGG |
| ATF7-F | TATGGGAGACGACAGACCGT |
| ATF7-R | TTGGGGTAACCTCCTCCACT |
| KDM2B-F | GACGCAAGCGGCTCAAAC |
| KDM2B-R | CTTCTTCTTCTCCTCCGGGC |
| KDM4A-F | GGCTTTGGGCTGTAGATTCCT |
| KDM4A-R | TCCGCTCGAGCTCTTCAAAC |
| PCGF6-F | TGTTCCACAGCCAGTCCCTT |
| PCGF6-R | TGCACGTCGGATTTCCCTTA |
| KDM5B-F | AGCCAAACCTGACCACAGAC |
| KDM5B-R | TTCTGGCTTCCGTTGTCTCC |
| KDM1A-F | CCAACGGACAAGCTGACACT |
| KDM1A-R | CATCACTTGGGGGATTCGCTT |
| KDM1B-F | ACTGCTATTAAGCCTGAGACC |
| KDM1B-R | GCTTTGCCACACTCATTGGG |
| KDM3A-F | TGAGCCACACAGACAGGTTG |
| KDM3A-R | GCCTGTTTGAACAAGGGCAG |
| E2F6-F | TGCCTTCGCCATGAATCCTT |
| E2F6-R | TCGGACTCCCAGTTTCGTTG |
| GAPDH-F | AATGGGCAGCCGTTAGGAAA |
| GAPDH-R | GCGCCCAATACGACCAAATC |
| ChIP1-F | GAGTCTCATTCTGTCGCCCAGG |
| ChIP1-R | GGAGGCTGAGGCAGGAGAATC |
| ChIP2-F | GATTCTCCTGCCTCAGCCTCC |
| ChIP2-R | AGCTCACGCCTGTAATCCCAACACT |
| ChIP3-F | GCATTTCTCTACCTACAAGTGGAG |
| ChIP3-R | CATTCCAGCTTGGGCGAC |
| si-RCOR1-F1 | GUGGAAGAUAAAGUCUUAUTT |
| si- RCOR1-R1 | AUAAGACUUUAUCUUCCACTT |
| si-KDM1A-F1 | CCACCGAGUUCACAGUUAUTT |
| si- KDM1A-R1 | AUAACUGUGAACUCGGUGGTT |
| si-KDM2B-F1 | GGGCAAAGAUUUCAACUAUTT |
| si-KDM2B-R1 | AUAGUUGAAAUCUUUGCCCTT |
| si-PCGF6-F1 | GCGCCUGAUUAAUCUCUCUTT |
| si-PCGF6-R1 | AGAGAGAUUAAUCAGGCGCTT |
| si-JMJD6-F1 | GUCCCUAAAUUCAACUAAATT |
| si-JMJD6-R1 | UUUAGUUGAAUUUAGGGACTT |
|  |  |
