# Supplementary material for: E2F6/KDM5C promotes SF3A3 expression and bladder cancer progression through a specific hypomethylated DNA promoter
Source: Cancer Cell Int. 2022 Mar 5;22:109. doi: 10.1186/s12935-022-02475-4 (PMC8897952; doi:10.1186/s12935-022-02475-4)
Supplement: Supplementary file 2 — Additional file 2: Fig. S1. Analyzed some genes positively related to SF3A3 expression in BC tissues from the TCGA database. [file 12935_2022_2475_MOESM2_ESM.docx]

Supplementary Figure


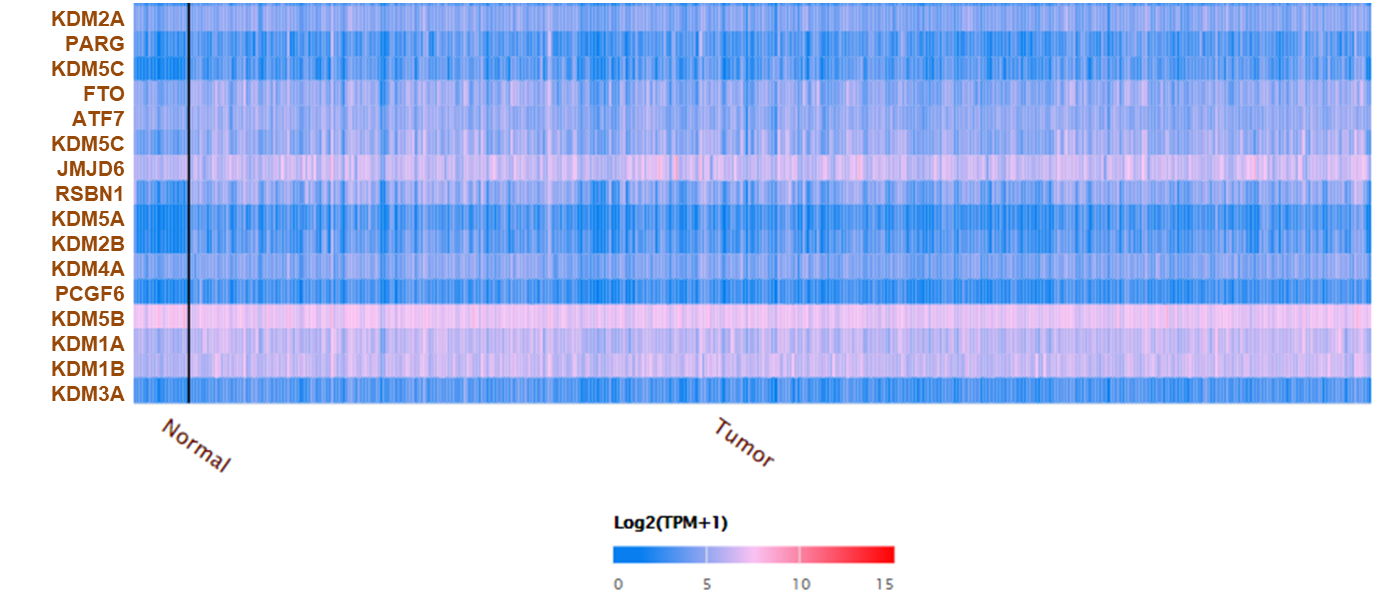


Supplementary Figure 1. Analyzed some genes positively related to SF3A3 expression in BC tissues from the TCGA database.
